# The association of sun-cured tobacco and cigarette use with global cognitive function, verbal fluency and memory in patients with chronic obstructive pulmonary disease: A cross-sectional study

**DOI:** 10.18332/tid/175973

**Published:** 2024-01-16

**Authors:** Xiaomei Chen, Jie Li, Jia Liu, Xiao Liu, Menghui Deng, Xunhu Dong, Yanni Yang

**Affiliations:** 1 School of Nursing, Army Medical University, Chongqing, China; 2 Department of Medicine, Qionglai Medical Center Hospital, Chengdu, China; 3 Department of Chemical Defense Medicine, School of Military Preventive Medicine, Army Medical University, Chongqing, China

**Keywords:** sun-cured tobacco, cigarette, cognitive decline, COPD

## Abstract

**INTRODUCTION:**

Some elderly people in China prefer sun-cured tobacco to cigarettes, and the composition of sun-cured tobacco and cigarettes is inconsistent. The influence of cigarettes on the cognitive function of COPD patients has been widely reported, but the research on sun-cured tobacco is relatively rare. Our study explored the association of sun-cured tobacco and cigarette use with cognitive decline in COPD patients.

**METHODS:**

This was a cross-sectional study. A total of 401 COPD patients were included, and 190, 103, and 108 participants were included in non-smoking, cigarette-smoking, and sun-cured tobacco groups, respectively. We evaluated the global cognitive function using the Beijing version of the Montreal Cognitive Assessment, verbal fluency function using an animal fluency test, and memory function using ten unrelated words.

**RESULTS:**

The participants of both cigarette-smoking (AOR=11.18; 95% CI: 1.28– 97.5) and sun-cured tobacco (AOR=10.46; 95% CI: 1.14–96.4) groups were more likely to develop mild cognitive impairment compared to the non-smoking group. The mean z scores of global cognitive function, verbal fluency, and memory were lower in cigarette-smoking and sun-cured tobacco groups than those in a non-smoking group; Multivariable linear regression showed that global cognitive function (β= -0.61; 95% CI: -1.04 – -0.18; and β= -0.48; 95% CI: -0.91 – -0.05) and verbal fluency (β= -0.79; 95% CI: -1.33 – -0.26; and β= -0.69; 95% CI: -1.23 – -0.16) of the sun-cured tobacco group and the cigarette-smoking group were significantly lower than those of the non-smoking group when adjusting for demographic and disease-related characteristics. However, there was no significant difference between the cigarette-smoking and sun-cured tobacco groups in global cognitive function, verbal fluency, and memory.

**CONCLUSIONS:**

Compared with non-smokers, the use of cigarettes and sun-cured tobacco may damage the cognitive function of COPD patients, especially in global cognitive function and verbal fluency.

## INTRODUCTION

Chronic obstructive pulmonary disease (COPD) is a common lung disease, and its incidence is increasing all over the world^1^. A cross-sectional study reported that the prevalence of mild cognitive impairment (MCI) was higher in COPD patients than in non-COPD patients^2^. A recent study reported that the incidence of cognitive decline in COPD patients was about 54%^3^. COPD with decreased cognitive function may reduce the treatment efficiency, not only affecting the physical function but also increasing mortality and disability^4^.

Smoking is a common risk factor for COPD, inhaling harmful substances contained in tobacco will reduce lung function and injure small airways and alveoli. Evidence suggests that the population-attributable risk for COPD from smoking is 51% globally. In addition, continuous smoking in COPD patients will aggravate the damage to lung function^5^. Moreover, smoking is independently associated with cognitive decline^6^. Although, several early studies reported that the acute intake of nicotine can improve cognitive function through nerve excitation^7,8^. However, a recent study indicated smoking is a common risk factor for cognitive function^9^, and the prevalence of cognitive impairment in smokers is significantly higher than that in non-smokers. One possible mechanism is that long-term intake can damage blood vessels, increase oxidative stress, and lead to cognitive decline^10^. Therefore, the high prevalence of cognitive impairment in patients with COPD may be related to smoking. However, most of the previous studies on smoking were about cigarettes, and few studies discussed the relationship between sun-cured tobacco and cognitive decline.

Sun-cured tobacco is planted by farmers and is a cultivated plant that belongs to handmade tobacco. Sun-cured tobacco can be divided into different types according to its shape, color, cultivation methods and so on^11^. Compared with cigarettes, many people prefer the taste and natural ingredients of sun-cured cigarettes. The diversity of natural conditions in China provides good conditions for the cultivation of sun-cured tobacco. Therefore, many provinces in China are rich in sun-cured tobacco, such as Sichuan, Jiangxi, Guangdong, and Heilongjiang. Different natural conditions, cultivation techniques, and sun-curing tobacco methods, have resulted in various types of sun-cured tobacco. The sales of this sun-cured tobacco have become the main source of the local economy, and sun-cured tobacco is very popular with people, especially the elderly^11^. Furthermore, a study confirmed that there were significant differences in the composition of cigarette and sun-cured tobacco^12^. Furthermore, the results of a 5-year prospective study showed that the proportion of male smokers of sun-cured tobacco was 75.75%, among which those aged 65–74 years were the most in the well-known sun-cured tobacco-producing area of Shifang City in China^13^. Combining the popularity of sun-cured tobacco and the difference with cigarettes, it was of practical significance to discuss the use of sun-cured tobacco. We speculated that sun-cured tobacco and cigarettes may have different cognitive effects on patients with COPD. However, the differences between them have not been discussed. Moreover, the cognitive decline of smokers is related to smoking status, duration, and type of smoking^14^. For COPD patients, the current research mainly explored the connection between the smoking status and duration on cognitive decline, and there are few studies that have mainly focused on the effects of different smoking types on cognitive function.

COPD patients performed poorly in cognitive tests to evaluate attention, memory, language, and executive function^15^, but smokers often suffer from memory loss and verbal fluency (reflecting executive function) disorder. In view of this, this study explored the association of sun-cured tobacco and cigarette use with the decline of global and specific cognitive functions (such as verbal fluency and memory) in COPD patients.

## METHODS

### Study design

This was a cross-sectional study, which was conducted from March 2022 to February 2023 in Sichuan Province, China. Sichuan Province is one of the provinces rich in sun-cured tobacco, and the planting scale of sun-cured tobacco in Sichuan is large, and its output and quality are among the best in the country.

### Sample size

We used the following formula to calculate the sample size^16^:


$$\text{n}=\frac{Z_{a/2}^{2}\times p\left(1-p\right)}{d^{2}}$$

with an estimated prevalence of 54%^3^ and 95% confidence interval, and a tolerance error of d=0.1, to obtain p=0.1×0.54=0.054. The required sample size was n=327, and it was increased by 10% to take into account non-response rate and sampling error. The sample size of this study was 360 individuals. About 401 participants were included in this study.

### Participants

**Figure 1:**
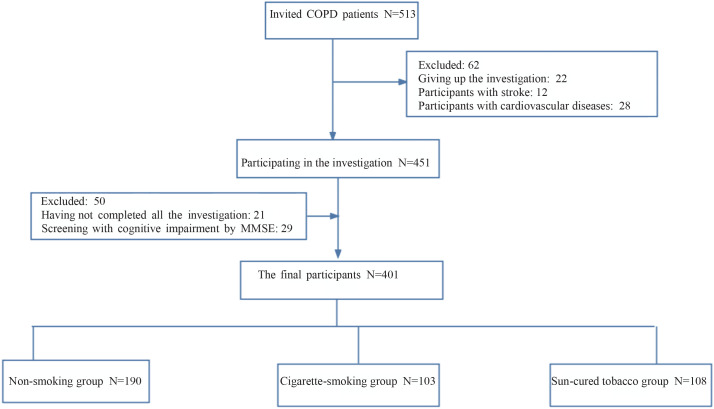
The procedure of recruitment for participants, survey on tobacco use among patients with COPD in Chengdu, 2022–2023 (N=401)

The inclusion criteria were: 1) diagnosed with COPD according to Global Initiative for Chronic Obstructive Lung Disease (2020 REPORT)^17^; 2) aged ≥40 years; and 3) having clear consciousness and being willing to give written informed consent. The exclusion criteria were: 1) unable to complete the investigation due to the serious illness; 2) having a mental illness; 3) having other diseases, including stroke and dementia; and 4) smoking both cigarette and sun-cured tobacco. The participant selection procedure is shown in Figure 1.

### Recruitment method

This study was divided into two stages to recruit participants. The specific recruitment procedures and precautions were detailed in another study^18^.

### Smoking and assessment

The questionnaire was used to assess the smoking status of participants. Participants who never smoked or have smoked <100 cigarettes in their lives were in the non-smoking group. Subjects who smoked at least one cigarette or sun-cured tobacco a day were divided into the cigarette-smoking group and the sun-cured tobacco group, respectively. Smoking quantity (number of daily cigarettes) and duration of smoking (years of smoking) were also evaluated in this study^19^.

Demographic characteristics (such as age, gender, education level, marital status, living arrangement, monthly household income, drinking habit, family history of dementia), factors associated with COPD [such as lung function index: percent predicted FEV1 (FEV1% pred) and smoking], were obtained at baseline from face-to-face interviews.

### Cognitive and lung function assessment

Cognitive function assessments were conducted by standardized, trained, and certified researchers and included measures of global cognitive function, memory, and verbal fluency. The global cognitive function was evaluated by the Beijing version of the Montreal Cognitive Assessment (MoCA), and the cut-off point of <26 was taken as the standard to identify an individual with MCI after excluding patients with dementia. MoCA includes eight cognitive domains: orientation, language, working memory, concentration, short-term memory, attention, executive function, and visuospatial ability^20^. The maximum score of MoCA is 30 points, and a higher score represents better cognition function^20^.

Ten unrelated words were used to evaluate memory, including immediate and delayed recall. The total score of the two items was 10, and the memory function was always divided into the sum of the two items. Higher scores indicated better memory function. Both of the two memory tests have good construct validity and consistency^21^.

An animal fluency test was conducted to evaluate verbal fluency. Participants were required to say as many animal names as possible within the 60s. The number of animals mentioned by participants was the score of this function. The verbal fluency test had well-documented reliability and validity^22^.

The percent predicted FEV1 (FEV1% pred) is an index to evaluate the severity of lung function, and GOLD1 to GOLD4 represent mild to extremely severe lung function injury^18^.

### Statistical analysis

We used SPSS Statistics 23.0 (IBM Corp, Armonk, NY, USA) for data analysis. Continuous variables were described as mean ± SD, and categorical variables as frequencies and percentages. We used ANOVA for continuous variables and the chi-squared test for categorical variables. Binary logistic regression was used to analyze the relationship between smoking type and MCI. Multivariable linear regression coefficients were used to examine the independent relationship among non-smoking group, cigarette-smoking group and sun-cured tobacco group in global cognitive function, memory and verbal fluency. The results were evaluated in three models. Model 1 was a univariate regression model, Model 2 was adjusted for demographic characteristics (such as age, gender, education level, marital status, living arrangement, monthly household income, family history of dementia, current drinker), and Model 3 was further adjusted for factors related to COPD [such as FEV1% pred, smoking quantity (cigarettes/day) and duration of smoking]. Before constructing the regression model, we checked the multicollinearity between covariates. A variance inflation factor (VIF) less than 10 means no multicollinearity. P-values were two-sided, and less than 0.05 were considered statistically significant.

## RESULTS

The characteristics of the participants are presented in Table 1. A total of 401 participants were included, and more than half were male (68.1%). One hundred ninety participants were non-smokers, and 103 and 108 participants were in the cigarette-smoking and sun-cured tobacco groups, respectively; 83.3% of the participants in the sun-cured tobacco group had less than 9 years of education, which was higher than that in the cigarette-smoking group (27.2%) and the non-smoking group (41.1%). The prevalence of MCI was 58.9%, and the prevalence of the sun-cured tobacco group (89.8%) was significantly higher than that of other groups.

**Table 1:** Population characteristics of a survey on tobacco use among patients with COPD in Chengdu, 2022– 2023 (N=401)

*Characteristics*	*Total (N=401) n (%)*	*Non-smoking group (N=190) n (%)*	*Cigarette-smoking group (N=103) n (%)*	*Sun-cured tobacco group (N=108) n (%)*	*p*
**Age** (years), mean ± SD	70.19 ± 10.20	67.37 ± 10.22	69.32 ± 11.62	75.98 ± 5.22	<0.001
**Gender**					<0.001
Male	273 (68.1)	66 (34.7)	101 (98.1)	106 (98.1)	
Female	128 (31.9)	124 (65.3)	2 (1.9)	2 (1.9)	
**Education level**					<0.001
Primary school and lower	196 (48.9)	78 (41.1)	28 (27.2)	90 (83.3)	
Junior middle school and higher	205 (51.1)	112 (58.9)	75 (72.8)	18 (16.7)	
**Marital status**					0.025
Married	338 (84.3)	165 (86.8)	83 (80.6)	90 (83.3)	
Divorced	29 (7.2)	15 (7.9)	11 (10.7)	3 (2.8)	
Widowed	34 (8.5)	10 (5.3)	9 (8.7)	15 (13.9)	
**Living arrangement**					0.114
Living alone	34 (8.5)	16 (8.4)	13 (12.6)	5 (4.6)	
Not living alone	367 (91.5)	174 (91.6)	90 (87.4)	103 (95.4)	
**Monthly household income** (RMB)					<0.001
<5000	104 (25.9)	35 (18.4)	12 (11.7)	57 (52.8)	
≥5000	297 (74.1)	155 (81.6)	91 (88.3)	51 (47.2)	
Current drinker*					<0.001
No	294 (73.3)	170 (89.5)	46 (44.7)	78 (72.2)	
Yes	107 (26.7)	20 (10.5)	57 (55.3)	30 (27.8)	
**Family history of dementia**					0.221
No	379 (94.5)	180 (94.7)	100 (97.1)	99 (91.7)	
Yes	22 (5.5)	10 (5.3)	3 (2.9)	9 (8.3)	
**Smoking quantity** (cigarettes/day)					0.535
<10	105 (49.8)	-	49 (47.6)	56 (51.9)	
≥10	106 (50.2)		54 (52.4)	52 (48.1)	
**Duration of smoking** (years)					0.110
<30	100 (47.4)	-	43 (41.7)	57 (52.8)	
≥30	111 (52.6)		60 (58.3)	51 (47.2)	
**FEV1% pred**					<0.001
GOLD1	105 (26.2)	69 (36.3)	27 (26.2)	9 (8.3)	
GOLD2	190 (47.4)	93 (48.9)	44 (42.7)	53 (49.1)	
GOLD3	100 (24.9)	27 (14.2)	30 (29.1)	43 (39.8)	
GOLD4	6 (1.5)	1 (0.5)	2 (1.9)	3 (2.8)	
**MCI**					<0.001
No	165 (41.1)	115 (60.5)	39 (37.9)	11 (10.2)	
Yes	236 (58.9)	75 (39.5)	64 (62.1)	97 (89.8)	
**Global cognitive function** (MoCA), mean ± SD	22.53 ± 3.84	24.02 ± 0.27	22.93 ± 0.32	19.54 ± 0.28	<0.001
**Verbal fluency,** mean ± SD	16.58 ± 6.31	18.71 ± 0.46	16.83 ± 0.57	12.58 ± 0.45	<0.001
**Memory**, mean ± SD	8.59 ± 2.34	9.21 ± 0.16	8.19 ± 0.27	7.90 ± 0.19	<0.001

MoCA: Montreal Cognitive Assessment. MCI: mild cognitive impairment.

* Current drinker defined as a person who drinks at least one glass of wine per week and lasts for 1 year and more. Smoking quantity (cigarettes/day) and duration of smoking (years) were just for smokers.

RMB: 1000 Chinese Renminbi about US$140.

For participants in the non-smoking group, the mean z scores of global cognitive function, verbal fluency and memory were the highest in the three groups, and the scores of the cigarette-smoking group were higher than that of the sun-cured tobacco group (0.39 vs 0.10 vs -0.78; 0.34 vs 0.04 vs -0.63; 0.26 vs -0.17 vs -0.29; all p<0.001) (Table 2).

**Table 2:** Global and specific cognitive function z scores and 95% confidence intervals in different groups, survey on tobacco use among patients with COPD in Chengdu, 2022–2023 (N=401)

*Variables*	*Non-smoking group (N=190)*	*Cigarette-smoking group (N=103)*	*Sun-cured tobacco group (N=108)*	p*
**Global cognitive function** (MoCA)	0.39 (0.25–0.52)	0.10 (-0.06–0.27)	-0.78 (-0.93 – -0.63)	<0.001
**Verbal fluency**	0.34 (0.19–0.48)	0.04 (-0.14–0.22)	-0.63 (-0.78 – -0.49)	<0.001
**Memory**	0.26 (0.12–0.39)	-0.17 (-0.39–0.06)	-0.29 (-0.45 – -0.13)	<0.001

* ANOVA was used to compare the differences in the z scores of the global cognitive function, verbal fluency, and memory among the three groups.

MoCA: Montreal Cognitive Assessment.

The results of multivariable logistic regression showed that in three models, the participants of the cigarette-smoking group and sun-cured tobacco group were more likely to suffer from MCI compared to the non-smoking group (OR=11.18; 95% CI: 1.28–97.5, p=0.029; and OR=10.5; 95% CI: 1.14–96.4, p=0.038 in Model 3) (Table 3).

**Table 3:** Multivariable binary logistic regression for MCI of three groups, survey on tobacco use among patients with COPD in Chengdu, 2022–2023 (N=401)

*Variables*	*Non-smoking group (75/190; 39.47%)*	*Cigarette-smoking group (64/103; 62.14%)*		*Sun-cured tobacco group (97/108; 89.81%)*	
		*OR (95% CI)*	*p*	*OR (95% CI)*	*p*
Model 1	®	2.52 (1.54–4.12)	<0.001	13.52 (6.79–26.91)	<0.001
		***AOR (95% CI)***		***AOR (95% CI)***	
Model 2	®	8.82 (3.26–23.86)	<0.001	6.57 (2.32–18.64)	<0.001
Model 3	®	11.18 (1.28–97.54)	0.029	10.46 (1.14–96.41)	0.038

Model 1: unadjusted. AOR: adjusted odds ratio. Model 2: adjusted for age, gender, education level, marital status, living arrangement, monthly household income, family history of dementia, current drinker. Model 3: adjusted as for Model 2 plus FEV1% pred, smoking quantity (cigarettes/day) and duration of smoking (years). ® Reference category.

Multivariable linear regression results showed that the z scores of both global cognitive function (β= -0.48; 95% CI: -0.91 – -0.05, p=0.028 vs β= -0.61; 95% CI: -1.04 – -0.18, p=0.005) and verbal fluency (β= -0.69; 95% CI: -1.23 – -0.16, p=0.011 vs β= -0.79; 95% CI: -1.33 – -0.26, p=0.004) were lower in cigarette smokers and sun-cured tobacco smokers compared to non-smokers in Model 3, and the z scores of the sun-cured tobacco group were the lowest. However, the association with memory loss was not significant in either Model 2 (p=0.055) or Model 3 (p=0.707) in the sun-cured tobacco group (Table 4).

**Table 4:** The associations of sun-cured tobacco, cigarette and non-smokers with global and specific cognitive function z scores, survey on tobacco use among patients with COPD in Chengdu, 2022–2023 (N=401)

*Variables*	*Non-smoking group (N=190)*	*Cigarette-smoking group (N=103)*		*Sun-cured tobacco group (N=108)*	
		*β (95% CI)*	*p*	*β (95% CI)*	*p*
**Global cognitive function** (MoCA)					
Model 1	®	-0.28 (-0.49 – -0.07)	0.008	-1.17 (-1.38 – -0.96)	<0.001
Model 2	®	-0.52 (-0.73 – -0.31)	<0.001	-0.65 (-0.89 – -0.42)	<0.001
Model 3	®	-0.48 (-0.91 – -0.05)	0.028	-0.61 (-1.04 – -0.18)	0.005
**Verbal fluency**					
Model 1	®	-0.29 (-0.52 – -0.08)	0.008	-0.97 (-1.19 – -0.75)	<0.001
Model 2	®	-0.56 (-0.82 – -0.31)	<0.001	-0.66 (-0.94 – -0.38)	<0.001
Model 3	®	-0.69 (-1.23 – -0.16)	0.011	-0.79 (-1.33 – -0.26)	0.004
**Memory**					
Model 1	®	-0.42 (-0.66 – -0.19)	<0.001	-0.55 (-0.78 – -0.32)	<0.001
Model 2	®	-0.49 (-0.78 – -0.19)	0.001	-0.31 (-0.64–0.01)	0.055
Model 3	®	-0.04 (-0.65–0.56)	0.891	-0.12 (-0.49–0.72)	0.707

Statistical analysis: multivariable linear regression analysis. Model 1: unadjusted. Model 2: adjusted for age, gender, education level, marital status, living arrangement, monthly household income, family history of dementia, current drinker Model 3: adjusted as for Model 2 plus FEV1% pred, smoking quantity (cigarettes/day) and duration of smoking (years). MoCA: Montreal Cognitive Assessment. ® Reference category.

The results of multivariable linear regressions of cognitive function between the sun-cured tobacco group and cigarette-smoking group showed that participants in cigarette-smoking group had better global cognitive function (β=0.97; 95% CI: 0.73–1.21) and verbal fluency (β=0.75; 95% CI: 0.50–1.01) compared with those in the sun-cured tobacco group in Model 1. However, in Models 2 and 3, participants in the cigarette-smoking group and sun-cured tobacco group had no significant association between global cognitive function, verbal fluency, and memory (Table 5).

**Table 5:** Comparison of global and specific cognitive function z scores between sun-cured tobacco group and cigarette group, survey on tobacco use among patients with COPD in Chengdu, 2022–2023 (N=401)

*Variables*	*Sun-cured tobacco group (N=108)*	*Cigarette-smoking group (N=103)*	
		*β (95% CI)*	*p*
**Global cognitive function** (MoCA)			
Model 1	®	0.97 (0.73–1.21)	<0.001
Model 2	®	0.08 (-0.18–0.34)	0.542
Model 3	®	0.08 (-0.17–0.33)	0.520
**Verbal fluency**			
Model 1	®	0.75 (0.50–1.01)	<0.001
Model 2	®	0.23 (-0.10–0.56)	0.174
Model 3	®	0.24 (-0.09–0.56)	0.149
**Memory**			
Model 1	®	0.12 (-0.14–0.36)	0.396
Model 2	®	0.14 (-0.21–0.49)	0.429
Model 3	®	0.13 (-0.22–0.48)	0.462

Model 1: unadjusted. Model 2: adjusted for age, gender, education level, marital status, living arrangement, monthly household income, family history of dementia, current drinker. Model 3: adjusted as for Model 2 plus FEV1% pred, smoking quantity (cigarettes/day) and duration of smoking (years). MoCA: Montreal Cognitive Assessment. ® Reference category.

## DISCUSSION

To our knowledge, this was the first study in China to investigate the association of sun-cured tobacco and cigarette use with the decline of global and specific cognitive function in COPD patients. In the unadjusted model, the z score of the global cognitive function of the sun-cured tobacco group and the cigarette-smoking group was significantly lower than that of the non-smoking group. After adjusting for the demographic and disease-related confounders, the z scores of global cognitive function of the sun-cured tobacco group and the cigarette-smoking group were still significantly lower than those of the non-smoking group, which was contrary to previous research. For example, a study demonstrated that acute nicotine intake was associated with cognitive benefit^23^. In addition, a meta-analysis showed that smoking could improve cognitive function^24^. However, the negative impact of smoking on cognition was also increasingly confirmed. Our findings are similar to a case-control study, in which, the incidence of MCI in the elderly who smoke was 3.04 times higher than never smokers (OR=3.04; 95% CI: 1.45–6.35)^6^. Some longitudinal studies and observational studies also indicated that smoking might increase the risk of cognitive decline^25,26^. The different effects of nicotine intake and smoking on cognitive function may be related to the content and duration of nicotine intake in tobacco. A meta-analysis showed that elderly smokers in the general population were at higher risk of cognitive decline than non-smokers^27^. However, the risk of cognitive decline was significantly higher for people with COPD than for the general population, and this difference is particularly pronounced among smokers^28^. This is similar to our findings. There are several possible mechanisms to explain the link between smoking and cognitive decline. The composition of tobacco includes some toxicants and some neurotoxic compounds that affect the nervous system, which could lead to the decline of cognitive ability of the elderly^29^. In addition, the decline of cognitive function caused by smoking is mainly related to the lesions of periventricular and subcortical white matter. Another mechanism that could explain the association between smoking and cognitive decline is lung function. As is well known, smoking is an important risk factor for lung injury and COPD. A previous study reported independent links between lung function and cognitive decline^30^, and poor lung function was associated with poor cognitive function. Therefore, smokers in COPD patients may be more likely to have cognitive decline.

In this study, apart from global cognitive function, the z scores of verbal fluency and memory remained significantly lower in the sun-cured tobacco group and cigarette-smoking group than in the non-smoking group. However, after adjusting for demographic and disease-related factors, the results of multivariable linear regression showed that there were no significant differences in memory. However, when only adjusting for the demographic confounders, the memory of the cigarette-smoking group was significantly lower than that of the non-smoking group, but there was no significant correlation between the sun-cured tobacco and non-smoking groups. The result of this study was similar to a cohort study, in which the current smokers had a greater decline in global cognitive function and executive function in the past 10 years than those who never smoked. The negative influence of smoking was greater on executive function than memory^9^. However, one meta-analysis revealed that nicotine could enhance cognitive function in multiple domains, such as memory^24^. The quantity and duration of nicotine intake might cause this inconsistency.

Compared to the non-smoking group, the global cognitive function and verbal fluency of the sun-cured tobacco group and cigarette-smoking group were significantly lower, and the decline in the sun-cured tobacco group was even greater (-0.61vs -0.48), but there was no significant difference in memory. The differences in cognition between sun-cured tobacco and cigarette use in COPD patients have never been compared in previous studies. However, the relationship between smoking use of combustible cigarettes, e-cigarettes and e-liquid, and passive smoking, and the incidence of subjective cognitive decline in Korean adults has been discussed. The results of that study showed that different types of smoking may lead to different incidences of subjective cognitive decline^31^. Sun-cured tobacco has a long history in Sichuan, China and is widely planted in all parts of Sichuan, and is especially popular among elderly men. According to a previous investigation, sun-cured tobacco and cigarettes were popular among COPD patients in Sichuan Province. The contents and components of cigarettes and sun-cured tobacco differed due to the differences in manufacturing and storage conditions^32^. An early prospective study showed that the use of sun-cured tobacco was related to mortality from cerebrovascular disease, tumors, cardiovascular disease, and respiratory disease. Therefore, the association of sun-cured tobacco and cigarette use with cognition may differ.

### Limitations

A few limitations of our study should be illustrated. Firstly, this was a cross-sectional study, from which it is impossible to determine the causal relationship between smoking and cognition. Secondly, despite all the efforts to control some confounding factors, there were still some potential confounding factors that have not been taken into account. Thirdly, this study did not include smoking cessation as a covariate but used smoking quantity and duration of smoking instead. Fourth, the variables on smoking were all self-reported by participants and convenient sampling was used in this study, which may lead to bias. Finally, limited generalizability to other countries could be included in our study.

## CONCLUSIONS

Compared with non-smokers, cigarettes and sun-cured tobacco may damage the cognitive function of COPD patients, especially in global cognitive function and verbal fluency. While advising COPD patients to quit smoking and paying attention to the harm of smoking to human health, we should not only pay attention to cigarettes but also pay attention to users of sun-cured tobacco.

## Data Availability

The data supporting this research are available from the authors on reasonable request.
